# Bayesian analyses question the role of climate in Chulmun demography

**DOI:** 10.1038/s41598-021-03180-4

**Published:** 2021-12-10

**Authors:** Habeom Kim, Gyoung-Ah Lee, Enrico R. Crema

**Affiliations:** 1 Department of Anthropology, University of Oregon, 1321 Kincaid Street, Eugene, OR 97403 USA; 2 Department of Archaeology, University of Cambridge, Cambridge, CB2 3ER UK

**Keywords:** Archaeology, Archaeology, Biological anthropology

## Abstract

We investigate the relationship between climatic and demographic events in Korea during the Chulmun period (10,000–3,500 cal. BP) by analyzing paleoenvironmental proxies and ^14^C dates. We focus on testing whether a cooling climate, and its potential negative impact on millet productivity around the mid 5th-millennium cal. BP, triggered the population decline suggested by the archaeological record. We employ a Bayesian approach that estimates the temporal relationship between climatic events and change-points in the rate of growth in human population as inferred from radiocarbon time frequency data. Our results do not support the climate-induced population decline hypothesis for three reasons. First, our Bayesian analyses suggest that the cooling event occurred after the start of the population decline inferred from the radiocarbon time–frequency record. Second, we did not find evidence showing a significant reduction of millet-associated dates occurring during the cooling climate. Third, we detected different magnitudes of decline in the radiocarbon time–frequency data in the inland and coastal regions, indicating that the even if cooling episodes were ultimately responsible of these population ‘busts’, their impact was most likely distinct between these regions. We discuss our results highlighting the long tradition of mobility-based subsistence strategy in coastal regions as a potential factor contributing to the regional differences we were able to detect.

## Introduction

Climate change has long been at the centerpiece of explanatory models of prehistoric demographic changes^1–5^. The last decade witnessed a renewed interest in this topic due to the ongoing climate crisis and the new opportunities offered by the increasing availability of paleoenvironmental proxies and large ^14^C datasets used as demographic proxies^6,7^. The latter, in particular, has benefited from a substantial growth in the number of regional case studies, most typically involving the use of the summed probability distribution of calibrated ^14^C dates (hereafter SPDs). Based on the assumption of a consistent correlation between the number of dates and the human population size^8^, SPDs are now commonly utilized to reconstruct past demographic trajectories and their relationship to climate changes, from simple visual comparisons of time-series to more complex statistical analyses and models^9–16^.

While these studies undoubtedly reveal a more nuanced picture of past human-climate relationships, SPDs, and more specifically, the assumption of ‘more dates–more people,’ have also raised criticisms. These range from the interpretability of the proxies in relation to formation, taphonomic, and recovery processes to the wide range of statistical biases associated with the summation of ^14^C dates^17–20^. One such issue is the intrinsic uncertainty associated with these curves that arises from a combination of different errors, namely, sampling, radiometric measurement, and calibration. As a result, it has been noted that visual inspections of SPD values should be carried out with caution, and more importantly, ‘direct’ statistical analyses of SPD values should be avoided as they do not account for the different forms of uncertainties associated with the ^14^C data^19,21,22^.

Environmental proxies deployed in statistical comparisons with radiocarbon time-frequencies are also affected by their own forms of chronological uncertainties. These are generally accounted for in age-depth models but rarely integrated as part of final correlative analyses. As a result, the temporal relationship between particular trends in the demographic and the paleoenvironmental proxies (e.g. a population drop vs a cooling event) are never formally evaluated. The consequence of this lack of formality is observed when temporal proximity of climatic and demographic signatures is assumed to be sufficient to argue for a specific order—most typically the climatic event occurring before the demographic one—and ultimately interpreted as evidence of a causal relationship. While to some extent such a prior belief can be justified, the extent of chronological uncertainties still needs to be accounted for, particularly if we wish to evaluate models that consider not just the temporal order but also the temporal distance between these events. The latter can reveal far more insights about adaptive responses and time-lags rather than simplistic models of climate-induced demographic processes (see for examples^10,11^).

This study investigates the relationship between climatic and demographic events during the Chulmun period (10,000–3,500 cal. BP) in Korea by taking into account these specific challenges. Chulmun, referring to the incised decoration pottery, is a local term for the Neolithic period, and is typically divided into six subphases: Incipient (10,000–8,000 cal. BP), Initial (8,000–6,500 cal. BP), Early (6,500–5,500 cal. BP), Middle (5,500–5,000 cal. BP), Late (5,000–4,000 cal. BP), and Final (4,000–3,500 cal. BP). Taking advantage of a wealth of ^14^C dates accumulated over recent decades due to the increasing number of rescue excavations in Korea, several authors have already attempted to infer the population dynamics of the Chulmun period using SPDs^23–26^. These studies mark an important juncture in Korean archaeology as pioneering studies that intensively utilized large available ^14^C datasets to reconstruct population trends during the Chulmun. Although each of these works have employed slightly different selection criteria for the ^14^C datasets used to generate their SPDs and sometimes integrated additional population proxies such as pithouse and settlement counts (see for example^24^), they all agree on the existence of a major population boom during the Middle Chulmun, followed by a population bust towards the Late and Final Chulmun periods.

This study specifically focuses on the latter phenomenon, the population decline phase during the Late and Final Chulmun. Typically, the cause of this decline is attributed to the cooling climate conditions occurring around the mid-5th millennium cal. BP. According to Ahn et al.^24^ and Ahn and Hwang^26^, the Chulmun population peaked by the Middle Chulmun due to the adoption of millet cultivation and sedentary lifestyle originated from the central-western inland region. However, the onset of cooling climate by the end of Middle Chulmun severely decreased the productivity of plant resources, particularly cultivated millets such as foxtail millet (*Setaria italica*) and broomcorn millet (*Panicum miliaceum*). Once millets stopped being a viable subsistence resource due to the deteriorating climate condition, Chulmun people abandoned their cultivation as well as the sedentary lifestyle, thereby triggering the observed population decline toward the Late and Final Chulmun. The evidential ground of this hypothesis rests on the two premises. Multiple paleoclimate proxies measured near and within the Korean peninsula indicate that a global scale cold/dry climate also occurred in Korea between 5,500 and 4,500 cal. BP. The number and scale of Chulmun settlements in many regions of Korea decreased in the Late/Final phase^24^.

Previous Chulmun population studies share a number of methodological shortcomings that make the evaluation of this hypothesis difficult. First, their methods of investigation are largely limited to the visual inspection of the trends in ^14^C data histograms and SPDs. As far as we are aware, Oh et al.^25^, is the only study that applied statistical testing to examine SPDs of the Korean peninsula, although their objective was simply to determine whether the inferred demographic curve deviated from an exponential growth. Second, while such a null hypothesis significance testing (NHST) approach can highlight interesting fluctuations in the inferred demographic trajectories, it does not directly allow us to evaluate the potential impact of particular climatic events. Third, these studies do not incorporate spatial perspectives into the overall interpretations of the ^14^C data, thereby contributing to a unilinear narrative on the processes leading to population changes. In doing so, they assume a ubiquitous process of population decline without considering the possibility that the population trend may vary by region. Fourth, despite the critical role of the decline of millet cultivation in this hypothesis, extant studies do not integrate ^14^C data on charred millets in their assessment of Chulmun population trends.

Here we test the hypothesis that the population decline observed during this period was climate-induced by addressing some of the methodological gaps of previous studies. We use a ^14^C dataset (n = 683) from Chulmun sites and compare its variation in time–frequency against local paleoclimatic data and dates associated with charred millet grains or their impression on pottery, whilst taking into account possible variations in trends between coastal and inland regions. If the hypothesis on a climate-induced population decline is supported, we expect to observe the following three patterns. First, the cooling climatic event would precede the population decline. Second, the population decline would be coupled with a significant decline in the relative frequency of ^14^C dates associated with millets. The decline of millet frequency would suggest that the population decline is closely tied with the failure of millet cultivation. Third, the rate and the timing of decline would be similar across inland and coastal regions. A regionally undifferentiated pattern of decline would validate the assumption that the hypothesized process leading to the population decline is applicable on a macro-regional scale.

**Figure 1:**
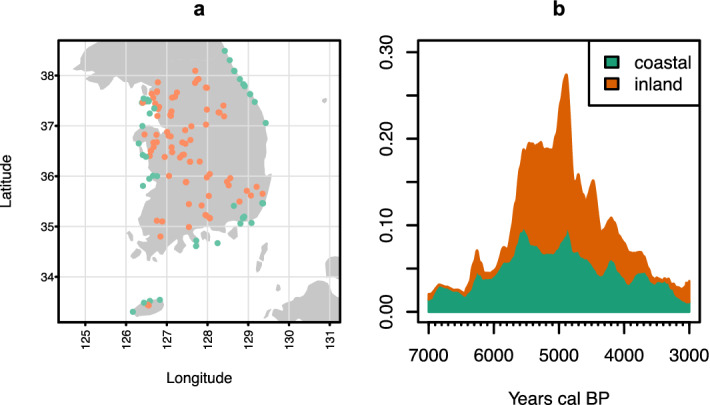
Distribution of sampling location of ^14^C dates (**a**) and summed probability distribution of the coastal and inland regions (**b**). Figure generated using R v4.1.0 (https://www.r-project.org/), basemap from GADM (https://gadm.org/).

To evaluate these expectations, we first divided our ^14^C dataset into two regional groups, coastal and inland (Fig. 1). The SPD of the two regions is in line with the pattern demonstrated in the previous studies, which indicated a pattern of population growth toward the Middle Chulmun, followed by a decline in the Late/Final phase.

This regional grouping is chosen in recognition that the subsistence practices in the two regions were different prior to the Middle Chulmun. Chulmun occupations on the coast exhibited a long history of mobility-based strategy, consisting of residential mobility, where hunter-gatherer groups seasonally moved from one locality to another for subsistence practices (sensu Binford^27^). The inland region, on the other hand, showed a strong tradition of large-scale sedentism that emerged in the Early Chulmun and intensified toward the Middle Chulmun^24,28^. We consider the possibility that the mobile hunter-gathering tradition, prevalent in the coastal regions before the Middle Chulmun, may have lingered and contributed to regional variations in the population decline pattern toward the Late/Final phase. To check this possibility, we fit a truncated double-exponential hierarchical Bayesian model^22^ to the ^14^C frequency data of the two regions. Fitted parameters of the double-exponential model provide the estimated rates of growth in the two regions, as well as the timing of when the population trend reversed from positive to negative growth rates. By comparing the fitted parameter to the timing of the abrupt cooling event based on two pollen sequences^29,30^ and the alkenone-based sea surface temperature reconstruction^31^, we aim to obtain a probabilistic estimate of the temporal order between a demographic decline and a cooling event. Finally, we compare the SPDs of all Chulmun dates against that of the dates associated with millet using the Mark-permutation test^32^ to determine whether and when the relative frequency of millet-associated dates changed over our window of analyses.

## Methods

### ^14^C dates

We collated an initial dataset comprising 894 ^14^C dates from 163 archaeological sites in South Korea and analyzed a subset of 683 dates from 136 sites after removing samples with an unknown origin, ones dated on marine specimens, and those with ^14^C age outside the bracket 6,200–2,800. We further separated our dataset into two groups based on the sampling location of the dates: *coastal* (n_dates_ = 363, n_sites_ = 51) when < 2 km from the current coast; and *inland* (n = 319, n_sites_ = 85) when > 2 km from the current coast (Fig. 1). The sample also comprises 45 dates associated with broomcorn or foxtail millet (Supplementary Fig. S2-S3).

### Age-depth models

We re-fitted age-depth models (Supplementary Fig. S4-S6) on three recently published paleo-climatic proxies based on the ratio of arboreal and total pollen (AP/TP)^29,30^ and alkenone-derived sea surface temperature reconstruction^31^. For each dataset we used Bayesian nonparametric Compound Poisson-Gamma model^33^ with IntCal20^34^ and Marine20^35^ calibration curves, the latter with locally obtained reservoir corrections (see Supplementary materials for details). For each proxy, we visually identified (Fig. S7) the start point and the endpoint of the largest drop in the estimated sea-surface temperature^31^ (d_1_ and d_2_), AP/TP^29,30^ (f_1_ and f_2_; g_1_ and g_2_) and then inferred their dates using posterior samples (Fig. S8).

### Summed probability distribution analysis

Probability distributions associated with calibrated ^14^C dates were aggregated to generate summed probability distributions (SPD). In order to take into account arbitrary and idiosyncratic definitions of sites as well as to reduce the potential impact of inter-site variations in dating intensity, a spatial and temporal binning procedure was carried out before generating the curves. The former was achieved by identifying spatial clusters of archaeological sites using the DBSCAN algorithm^36^ with ε = 1 km and minPts = 1. Temporal binning was carried out by grouping dates within the same cluster using the *binPrep* function of the *rcarbon* package^20^ using a temporal threshold (*h*) set to 100 years. Dates were calibrated using the IntCal20 calibration curve^34^, and aggregated to sum to unity within each cluster-bin before summation. Three SPDs were generated, one for each region (i.e. coastal and inland sites) using the binning procedure described above and one for dates associated with millets but without spatial and temporal binning.

SPDs of coastal and inland sites were compared using a mark-permutation test^32^ with a time range set to 7,000–3,000 cal. BP, and with 1,000 replicates to compute the global p-value (Fig. S1). The same method and settings were used to statistically assess whether the relative proportion of 14C dates associated with millets (n_total_ = 682, n_millets_ = 45; 6.59%) changed over time.

### Bayesian estimates of growth rate and change point

We fitted a truncated double-exponential growth model^22^ using the *nimbleCarbon* package^37^ to estimate the growth rates and identify the start-point of the decline in the density of ^14^C dates between 7,000 and 3,000 cal. BP. The truncated double-exponential growth model is defined by the following probability mass function:

$${p}_{t=a-i}=\frac{{(1+r)}^{i}}{{\sum }_{i=0}^{a-b}{(1+r)}^{i}}$$

$$r=\left\{\begin{array}{c}{r}_{1}\,\,\,\,\,\,\,  \text{if}\,\,t>c \\ {r}_{2}\,\,\,\,\,\,\,  \text{if}\,\, t\le c \end{array}\right.$$

where *p*_*t*_ is the probability of sampling a ^14^C date from time $t$ (in cal. BP), $r$ is the growth rate (equal to ${r}_{1}$ before the change point $c$ and to ${r}_{2}$ afterwards), and $a$ and $b$ are boundary parameters so that ${p}_{t<b}={p}_{t>a}=0$. To account for the potential impact of inter-site variation in sampling intensity we selected the ^14^C date with the smallest error for each spatio-temporal bin, and excluded all dates with cumulative calibrated probability below 0.5 between 7,000 and 3,000 cal. BP. The resulting thinned dataset consisted of 197 dates for the coastal sites and 179 dates for the inland sites. We treated the boundary parameters as constant ($a$ = 7,000 cal. BP; $b$ = 3,000 cal. BP), and used the following priors for our three parameters of interest:

$${r}_{1}\sim Normal\left(\mu =0,\sigma =0.0004\right)$$

$${r}_{2}\sim Normal\left(\mu =0,\sigma =0.0004\right)$$

$$c\sim TruncatedNormal(a=3000,b=7000,\mu =5000,\sigma =1000)$$

Which ensured both positive and negative growth rates within ranges typically observed in SPDs (cf Zahid et al.^38^), and a weakly informative prior for the change point with a slightly lower probability of occurrence in the boundary of the window of analysis. Prior predictive checks were carried out to examine the choice of prior parameters (Fig. S9).

Posterior samples of the parameters ${r}_{1}$, ${r}_{2}$, and $c$ were obtained using MCMC via the *nimble* package^39^, using for each data set (coastal, inland, and a combined set) three chains of 100,000 iterations, a burn-in of 10,000 steps, with the thinning parameter set to 6. Convergence and mixing of the chains were examined using trace plots, Gelman-Rubin diagnostic^41^, and effective sample sizes (Fig. S11, Table S5). Posterior predictive checks were carried out by comparing the observed SPDs (using the thinned dataset) against envelopes of 500 SPDs generated from growth models based on posterior parameters (Fig. S13).

### Measuring temporal relation between demographic and climatic events

The temporal order and distance between the change points in growth rates of each data set (*c*_inland_, *c*_coastal_, and *c*_all_) and the start point (*d*_1_, *f*_1_, and *g*_1_) and end point of abrupt declines (*d*_2_, *f*_2_, and *g*_2_) identified in the age-depth models, were computed by randomly sampling 5,000 values from the posterior distributions of relevant parameters and computing the pair-wise difference in the so-obtained calendar years.

## Results

### Regional variations in Chulmun population trajectory: Coastal versus Inland

**Figure 2:**
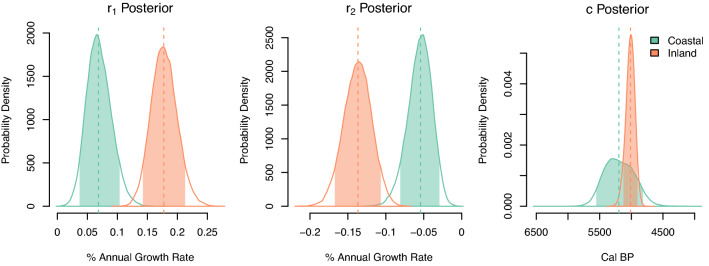
Marginal posterior distribution of growth rates *r*_*1*_ and *r*_*2*_ and change-point *c* for the inland and coastal radiocarbon dates. Highlighted regions depict the 90% higher posterior density interval. Figure generated using R v4.1.0 (https://www.r-project.org/).

Figure 2 (see also Fig. S12) shows the population growth and decline rates in the two regions before and after the change point of population trend as obtained by fitting the SPDs into the discrete double-exponential hierarchical Bayesian model. We define the change point as a point in time when the overall population trend reversed from a growth into a decline. The median growth and decline rates in the inland region were approx. 0.18% and -0.14%, whereas they were approx. 0.06% and -0.05% in the coastal region. The rate of past population changes in both regions is within the range of changes seen in other case studies on past populations^22,38,42^.

The result of our analysis opposes one of the expectations in the climate-induced population decline hypothesis. Despite its assumption in the ubiquitous applicability of the hypothesized process leading to the population decline, there is a clear pattern of spatially differentiated decline between the inland and the coastal regions (see also Fig. S1). During the decline phase after the change point, the population in the inland region decreases roughly at a rate 2.5 times greater than that of the coast. Also, the median posterior change point from the growing to declining phase indicates a delayed shift in the inland sites roughly by 200 years. We note, however, that the 90% high probability density (HPD) intervals overlap, primarily due to the higher uncertainty of the estimate for the coastal region (Fig. 2, see also Fig. S12). This overlap does not dismiss the possibility that the posterior changepoint in both regions is concurrent rather than distanced temporally.

### Cooling climate in relation to the timing of the change point

**Figure 3:**
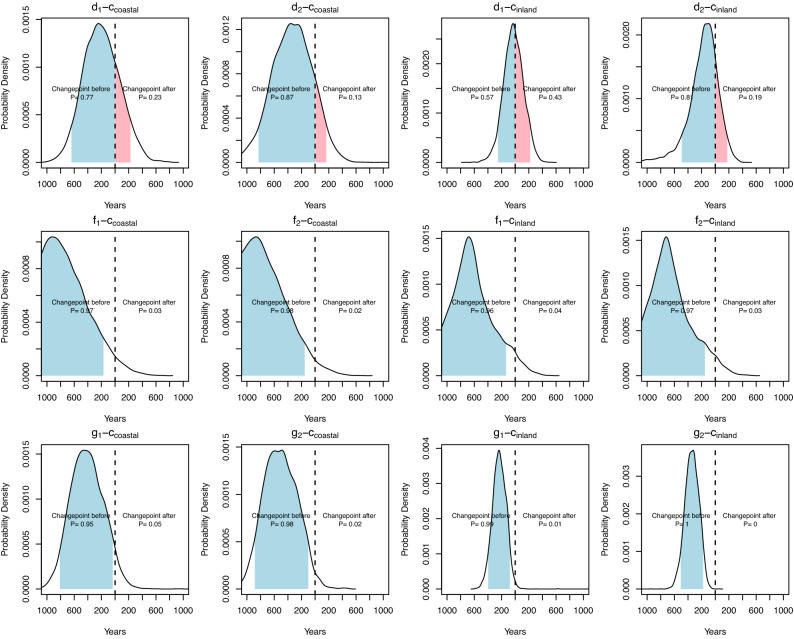
Temporal relationships between ^14^C density change points in in coastal (columns 1, 2) and inland regions (columns 3, 4) and climatic events based on drops in alkenone-derived sea-surface temperature from Kim et al.^31^ (d_1_, d_2_); and drops in arboreal to total pollen (AP/TP) ratios from Constantine et al.^30^ (f_1_, f_2_) and Park et al.^29^ (g_1_, g_2_). Subscript numbers represent the start (1) and endpoint (2) of the decline. Figure generated using R v4.1.0 (https://www.r-project.org/). See ESM for details.

The temporal relationship between the changepoints and the climatic events show some conflicting results. There is strong evidence that cooling events inferred from a drop in the ratio of arboreal to total pollen occurred *after* the changepoint observed in the frequency of ^14^C dates with probabilities systematically above 0.95 (events *f* and *g* in Fig. 3). The median posterior indicates a gap of 200 to 800 years between these events and the changepoints. In either case we were not able to detect no regional variations, with both inland and coastal regions showing a high probability that the decline in population occurred after the cooling in climate.

The alkenone-derived estimate of sea surface temperatures shows a much closer temporal distance between the cooling event and the changepoint in the fitted double-exponential model for the two regions (event *d* in Fig. 3). The 90% HPD of the difference in the dates cover both positive and negative values, hence suggesting that we cannot dismiss the possibility that the cooling occurred before the decline in the density of ^14^C dates. We also notice some differences in the temporal relationship between coastal and inland regions, with the probability that the beginning of the cooling event occurred after the population changepoint slightly higher in the former (event d_1_-C_coastal_ and d_1_-C_inland_ in Fig. 3).

The premise of the climate-induced population decline hypothesis rests on the cooling climate occurring before the population changepoint. Overall we did not find compelling evidence in support of such a relationship, and instead for two of the three paleoclimatic proxies our model actually suggest that the opposite (i.e. population decline occurring *before* cooling events) was more likely. The analysis based on the remaining paleoclimatic proxy based on the data from Kim et al.^31^ yielded an inconclusive result—neither confirming nor rejecting the expectation of the hypothesis.

### Mark-permutation test of millet-associated dates

**Figure 4:**
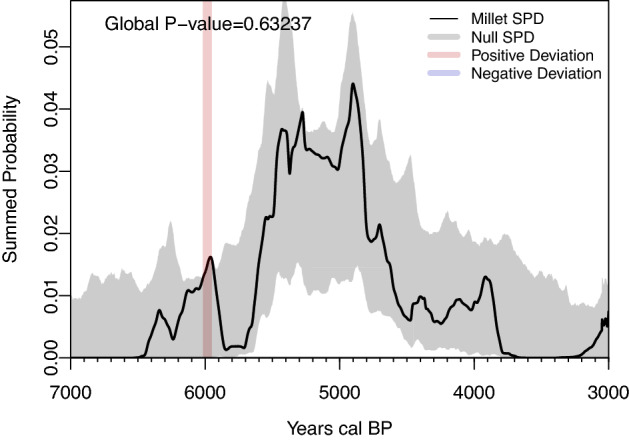
Mark permutation test of millet versus combined ^14^C dates from both regions. Figure generated using R v4.1.0 (https://www.r-project.org/).

The Mark-permutation test between dates associated with millets and all dates yielded a P-value of 0.63 indicating that the frequencies of millet-associated dates have not changed significantly relative to the overall frequency changes of the Chulmun absolute dates (Fig. 4). While it is worth taking into consideration the small sample size, the lack of significant reduction in the frequency of millet-associated dates does not support the claim that the failure of millet farming had occurred amidst the on-going climate changes in the Late Chulmun.

## Discussion

The combination of analyses we presented here provides sufficient evidence to question the narrative of cooling climate being the primary cause of the population decline observed during the Late and Final Chulmun periods. If the cooling climate has been directly responsible for triggering the Chulmun population decline, the population changepoint from positive to negative growth rates would have clearly occurred after a major cooling signal. However, our analyses were not able to determine that the cooling climate necessarily preceded the population changepoint. On the contrary, two out of the three paleoclimatic proxy data employed here indicate that the likelihood of the population changepoint prior to the cooling climatic event is much higher than the opposite. Our approach focused on the definition of these climatic events as significant drops in the paleoclimate proxy data. In doing so, we effectively treated climate deterioration as a one-time event, when effectively cooling climate might was a condition that most likely continuously impacted Chulmun communities for at least a few hundred years between 5,500 and 4,500 cal. BP. Nonetheless, the timing of the demographic and climatic events we have identified certainly raises questions on the existing narratives. Indeed, if the consequence of cooling was a deterioration of millet productivity, and this in turn was one of the contributing factors to the population decline, we should have been able to detect a significant decline in the millet-associated dates before or during the Late and Final Chulmun. However, our statistical test indicates that the frequency of millet-associated dates relative to all other ^14^C dates did not decrease significantly either in the Late or Final phase. While our statistical power was most likely small, this further questions the climate-induced population decline hypothesis.

Another factor adding weight against such a hypothesis is the ecology of millets. The dominant millet taxa during Chulmun, foxtail and broomcorn, are known to be highly tolerant species that can grow in various conditions including drought-prone cold environments. Both species are fast-growing grain crops, and can adapt to almost all types of soil conditions^43^. Broomcorn millet has one of the lowest water requirements of all cereals, requiring only an annual rainfall of 200–450 mm^44^. Foxtail millet can grow even less than 125 mm rainfall in semi-arid areas during the months of growth^45^. Broomcorn and foxtail can mature as short as 45 and 90 days respectively and have a large temperature tolerance. Such minimum requirements would have facilitated Early Holocene people in northern China to grow them without much effort as early as 10,000 cal. BP and spread widely throughout East Asia, Far Eastern Russia, and Europe^46,47^.

This study also finds that, while both inland and coastal regions experienced a general population decline in the Late/Final phase, the rate of decline was much greater in the inland region than the coast. This regional variability hints at the spatial processes at work during the population decline phase in the Late/Final Chulmun. The coastal region distinguishes itself from the inland region by its long tradition in mobile subsistence strategy. Here we briefly consider the possibility that this may have contributed to the observed regional variations in the population decline trend, particularly the moderate rate of decline in the coast compared to the inland.

The evidence for the residential mobility in the coastal region is found in some of the earliest Chulmun occupations. These occupations include occupations such as the Gosanri site in the coast of Jeju Island dating as early as 10,000 cal. BP, marking the earliest pottery culture in Korea^48^. The Gosanri site is believed to have been used as a task-specific site seasonally used for the lithic tool production^48^. Other occupations reflecting residential mobility are Osanri C and Munamri on the east coast dating to as early as 8,000 cal. BP, marking the start of the Initial Chulmun^49^. Almost concurrently, seasonal dwellings appeared along the southern coast as evidenced in the sites such as Tongsamdong, Bibongri, Beombang sites^49^. The Chulmun settlers in the coastal region utilized diverse seasonal resources of both marine and terrestrial catch, as well as millets, and probably used these sites as seasonal base camps rather than permanent settlements reflecting their residential mobility^28,48,50^. They practiced a ‘broad-spectrum economy’ procuring a diverse array of marine and terrestrial resources by hunting, gathering, fishing, and millet cultivation^50,51^.

The coastal community experienced a major subsistence transition onset of the Middle Chulmun. From the inland region of the central-western valley, a new subsistence tradition expanded to the rest of the peninsula, penetrating into the coast, in a phenomenon recognized by some as the ‘Middle Chulmun expansion^24,28^. While the new tradition is still largely grounded in the preceding phase’s broad-spectrum economy, it was newly paired with the beginning of large-scale sedentism. The most widely cited example of the new tradition is the Amsadong site in the Han River valley approximately 50 km inland from the coast. Amsadong yielded dozens of pit-houses and outdoor structures where food production and processing tools were found, including digging tools, reaping knives, and grinding slabs^52^. These sedentary communities are popularly regarded as food producers practicing millet cultivation^24,28^. However, the practice of small-scale food production was not confined to the new sedentary communities as the coastal communities also utilized plant resources, including millet and azuki bean, as early as the Early Chulmun^51^.

The conventional view on the Chulmun cultural trajectory accepts that the new sedentary way of life largely replaced the existing mobile tradition following the Middle Chulmun expansion. Indeed, this view seems to be supported by archaeological findings, including the ubiquitous popularization of the ‘comb-marked’ style pottery associated with the sedentary communities and new appearance of large-scale villages. This view also contends that the Chulmun villagers in the Late phase eventually abandoned the sedentism and readopted the a lifestyle consisting of logistical mobility mixed with some degree of residential mobility^24,26,53^. The evidence is attributed to the decline in the number of settlements and the increase of outdoor features, including open-air hearths and shell middens^24,26^.

The underlying assumption in this conventional account of Chulmun coastal communities is that they were passive adaptationists. They passively adopt new subsistence strategies in response to external factors such as the diffusion of new technology or the changes in climatic conditions while clearly breaking away from their old tradition. Instead of such an adaptationist account, we consider the possibility that the sedentary lifestyle popularized after the Middle Chulmun expansion did not completely replace the coast’s mobility-based subsistence practiced over hundreds of generations. This mobile strategy would have constituted an essential part of the coastal community’s traditional ecological knowledge (TEK). Coastal communities had accumulated and inherited their TEK, constructing a resilient cultural niche, and may have continued practicing mobility-based subsistence to some extent in the Late/Final phase even after the Middle Chulmun expansion.

TEK refers to a cumulative body of knowledge and practices about the relationships of living beings with one another and their physical environment evolved by adaptive processes and inherited by cultural transmission^54^. TEK may have provided relative resilience to the coastal communities by diversifying their subsistence strategies during a time when the overall population was declining due to the deteriorating sedentary way of life. TEK may therefore account for the reasons why the sharp decline observed in the inland regions during the Late/Final phase is not seen in the coast.

Ending the discussions on Chulmun population change, we point out that Chulmun is not a unique case where climate change does not play a causative role in triggering a population response. Many case studies worldwide reveal a nuanced relationship between the population and the climate, where the change in the former is impacted by the latter in a complex multitude of ways rather than in a deterministic one-way fashion. For example, Shennan et al.^9^ investigate the relationship between population and climate in Central and Northwest Europe during the mid-Holocene by computing cross-correlations between several climatic proxies and the SPDs. Their findings indicate that the variations in population levels are largely not associated with climatic changes. In a similar vein, DiNapoli et al.^55^ observe the population trend of Rapa Nui between the time of initial human settlement (ca. 800 cal. BP) and the period shortly after the European contact using Approximate Bayesian Computation method. Their result highlights the resilience of the island’s population, reflected in the steady population levels on the island until the timing of European arrival. This finding sharply contests the previous study’s claim that climate-induced drought resulted in large-scale societal disruption on the island. Another study that denotes a complex dynamic between the climate and population responses is Kelly et al.^10^. They examine the relationship between climate and population size over the past 13,000 years at Bighorn Basic in Wyoming through the Granger causality test. Their findings suggest that, while population level is closely linked with the changes in climatic conditions, climatic changes do not immediately translate to changes in the population level. Instead, they find a persistent 100–300 year time lag between a change in the moisture level and demographic responses. These studies remind us that the relationship between climate changes and demographic responses during the Chulmun period should be conceived beyond the terms of the cause-and-effect account.

## Conclusion

In this study, we examined the temporal relationship between climatic and demographic events during Chulmun period in Korea by analyzing paleoenvironmental proxies and 14C dates from Bayesian statistical approach. We particularly focused on testing the standing hypothesis, which posed the arrival of the cooling climate as the cause for the population decline toward Late/Final Chulmun. Our results do not support the climate-induced population decline hypothesis for three reasons. First, we could not determine that the cooling climatic event necessarily preceded the reversal point in population growth rate as inferred by the radiocarbon record. Second, we did not find evidence showing a significant reduction of millet-associated dates occurring during the cooling climate. Third, we detected a spatially differentiated pattern of decline in inland and coastal regions, where the rate of population decline is discernably more moderate in the coastal region compared to the inland, thus indicating that the cooling did not impact all populations equally.

We highlight TEK as a possible factor for the resilience of the population in the coastal region as reflected in the region’s relatively moderate rate of decline compared to the inland during the Late/Final phase. Our remark is based on the longue durée view of the Chulmun culture. By managing resources and knowledge that they inherited and passed down through hundreds of generations, coastal Chulmun people may have succeeded in sustaining their traditional ways of life despite the cultural and environmental changes that they encountered during the Late and Final phases. We believe this long-term view is critical to move forward the current discourse on Chulmun population trajectories away from the simple cause-and-effect explanations.

As a theoretical next-step, we suggest to emphasize the importance of the long-term view in the current discussions of Chulmun population changes. Current discussions on Chulmun population changes are often constructed in a unilinear adaptationist framework, where people are viewed as passive responders to external factors poised as the enforcing causes for cultural changes. It is undeniable that Chulmun people would have had to respond to various external impacts throughout their lives. However, such an adaptationist framework can overshadow the potential that the people’s long-term history had been actively shaping their responses to the various external factors and thereby impacting their cultural trajectory in complex ways and surrounding environments—a key premise of Traditional Ecological Knowledge Theory and Niche Construction Theory^56^.

## Supplementary Information

Supplementary Information.

## Data Availability

All raw data and R scripts required to reproduce the analyses presented in the paper can be found in the following dedicated GitHub repository: https://github.com/ercrema/NeolithicKoreaDemography. An archived copy can also be found on 10.5281/zenodo.5746633.
