# Supplementary material for: Bayesian analyses question the role of climate in Chulmun demography
Source: Sci Rep. 2021 Dec 10;11:23797. doi: 10.1038/s41598-021-03180-4 (PMC8664936; doi:10.1038/s41598-021-03180-4)
Supplement: Supplementary file 1 — Supplementary Information. [file 41598_2021_3180_MOESM1_ESM.pdf]

Supplementary Information for

Bayesian analyses question the role of climate in Chulmun demography

Habeom Kim, Gyoung-Ah Lee, Enrico R. Crema

1. Extended Dataset Description

We gathered a total of 894 ^{14}C dates from samples collected from Chulmun sites in South Korea. We built our ^{14}C date database using the previous compilation of the Chulmun dates made available to the scholarly communities by Chulmun specialists[1,2].

To ensure the scientific reproducibility and validity of the data used for analysis, we further applied the following filters to the collected dates. First, we eliminated samples of which we could not clearly identify its published primary source (i.e. site excavation reports). Second, we eliminated dates which were deemed too early or late to establish a reliable cultural association to the Chulmun period. More specifically we removed samples whose uncalibrated ^{14}C age were earlier than 6,200 BP or later than 2,800 BP. Third, we eliminated shell and animal bone dates, which contain potential marine signature, to control for the marine reservoir effect potentially impacting the result of our analysis. Finally for multi-component sites spanning multiple chronological periods, we only selected the dates with clear contextual association with a Chulmun feature to eliminate chronological ambiguity in dates. After these filtering, the total number of ^{14}C dates used for our analysis was 682. Of these, 640 (93.8%) were wooden charcoal, 29 (4.3%) various annual grains, and 13 (1.9%) uncharred wood. We further distinguished the dates that have contextual association with millet. We establish millet association to a date when the date itself was from a millet grain or was found in the same stratigraphic layer with confirmed presence of millet either by archaeobotanical or pottery impression evidence.

We then divide the ^{14}C datasets into two spatial groups: inland and coastal. We deemed a date to be coastal, if it was recovered from a site located within 2 km of the current coastal line. Otherwise, the date was placed into the inland group. To account for the sea-level change that occurred since the Chumun period, an exception was made if an inland site is known to have been located on the coast during the Chulmun occupations. The sites applicable to this exception were Bibongri, Sugari, Gadong, and Jukrimdong shell mounds sites in the Southeastern region of Korea[3,4,5,6]. These now-inland sites yielded artifacts and ecofacts of clear marine origins such as marine mammals, fish, and shells, thus the dates recovered from these sites are regarded as coastal.

2. Data Preparation for Summed Probability Distribution and Bayesian Analysis

We generated summed probability distributions of calibrated radiocarbon dates (hereafter SPD) of the coastal and inland regions between 7,000 and 3,000 cal. BP, using the *rcarbon* package[7] (v.1.4.1) of the R statistical computing language[8]. To account for potential biases arising from idiosyncratic definitions of archaeological sites (which often do not necessarily equate to past settlements) and inter-site variations in the sampling intensity of radiocarbon dates, we first grouped sites into spatial clusters using the DBSCAN algorithm[9], using a distance threshold ε equal to 1km and setting the minimum number of points per cluster

to 1, and subsequently generated temporal *bins*[10] using the *binPrep* function in *rcarbon* and setting a temporal distance (*h*) to 100 years. We thus define each spatio-temporal *bin* to be a group of radiocarbon dates that are close to each other in space and time. Both the visual display of the regional SPDs (Fig. 1, main text) and the mark-permutation test for assessing the difference in their shapes (Fig. S1, see below) were carried out by using as a basic unit of analyses these spatio-temporal *bins*. In both cases, radiocarbon dates from the same bin were calibrated using the IntCal20 calibration curve[11], and their resulting vector of probabilities summed and divided to the number of dates in the same bin (i.e. normalised to sum to unity).

The Bayesian model fitting procedure had a slightly different workflow. Firstly, to account for potential edge effects we excluded from our sample all dates with a calibrated cumulative probability within the time range of analyses (i.e. 7,000 - 3,000 cal. BP) below 0.5. Then we selected from each spatio-temporal bin the radiocarbon date with the smallest ^{14}C error (randomly selecting one in case multiple dates had the same ^{14}C error). Table S1 shows the number radiocarbon dates, *bins*, site clusters, and the sample used for Bayesian analyses for each region.

	**Number of ^{14}C Dates**	**Number of Sites**	**Number of DBSCAN clusters**	**Number of temporal bins**	**Sample size for Bayesian Analysis**
**Coastal**	363	51	42	201	197
**Inland**	319	85	76	183	179
**Total**	682	136	118	384	376

Table S1. Sample size for coastal and inland regions.

2. Mark Permutation Tests

3.1 Coastal vs Inland Site Dates

Temporal density of ^{14}C dates associated with coastal and inland sites were compared using mark permutation test[12] with 1,000 Monte-Carlo iterations to compute the local and global P-values. Results (Fig. S1) show significant departures from the null hypothesis of the two SPDs being samples from the same statistical population, with a Global P-value < 0.001.

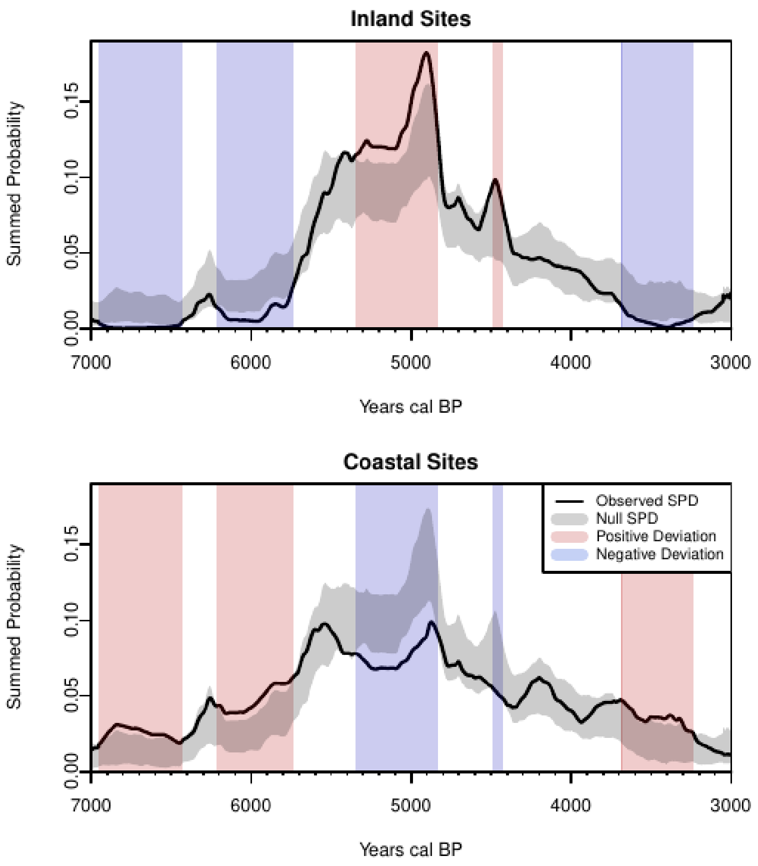

Figure S1 Mark permutation test between coastal and inland site.

3.2 Millet Dates

To assess the relationship between foxtail (*Setaria Italica*) and broomcorn millet (*Panicum miliaceum*) cultivation on the one hand and demography on the other, we extracted all radiocarbon dates associated with either crops from both regions (Fig. S2), generated an SPD (Fig. S3) and compared its shape to an SPD based on all radiocarbon dates via a mark permutation test[12] as implemented in the rcarbon R package[7]. The technique consists of comparing the observed SPD (in this case of millet dates, Fig. S1) against an envelope of simulated SPDs generated by randomly shuffling the marks associated with each date (in this case millet vs non-millet dates). Thus in practice, in this case the procedure is equivalent to generating SPDs by randomly sampling 45 radiocarbon dates from the pool of all radiocarbon dates available in the window of analysis. The null hypothesis is that there is no difference in the shape of the SPD based on millets vs the SPD based on all dates, which is equivalent to a stationary proportion of dates associated with the crop. SPD in this case were generated without spatio-temporal binning and the global P-value obtained via 1,000 permutations. The results (Fig. 4, main text) yielded a p-value of 0.69, suggesting that we cannot determine whether the rise and fall in the millet SPD is different from the overall fluctuations in the density of radiocarbon dates, and hence possibly human population dynamics.

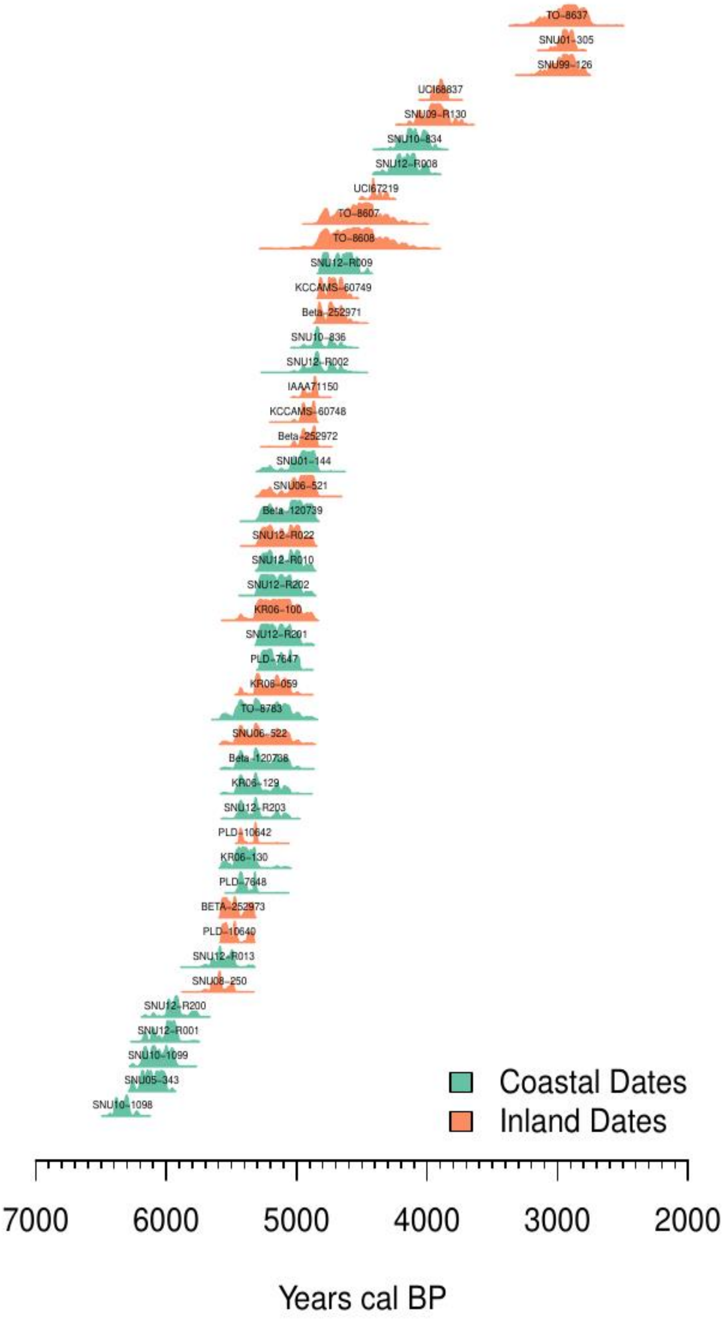

Figure S2 Calibrates dates associated with foxtail and broomcorn millets from coastal and inland sites (*n*=45).

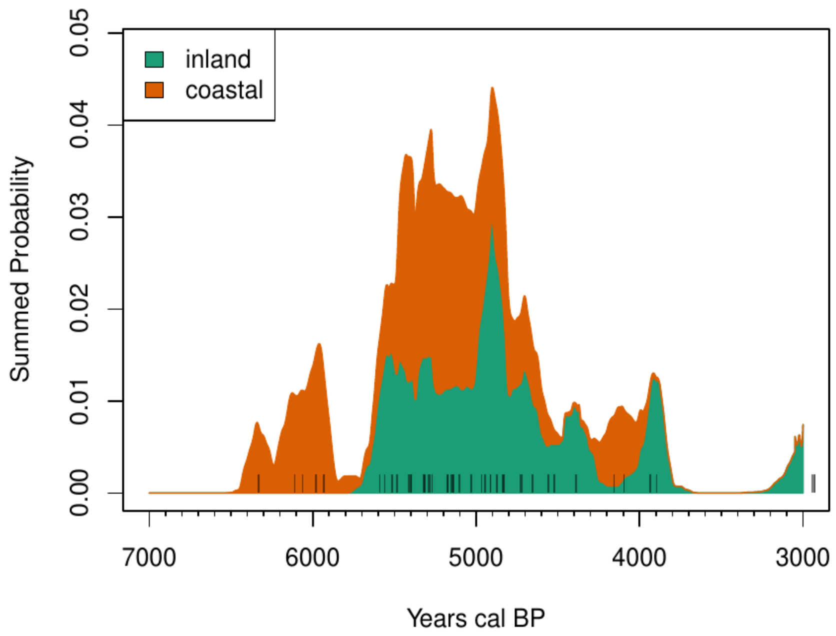

Figure S3 SPD of radiocarbon dates associated with either foxtail or broomcorn millet from coastal and inland regions. Vertical bars represent the median calibrated date of each specimen.

4. Age-Depth Modelling

We used a compound Poisson-Gamma model as implemented in the *Bchron* R package[13] to re-fit an age-depth model for the SSDP-102[14] (Fig. S4), GY-1[15] (Fig. S5), and Pomaeho[16] (Fig.S6) sediment cores using the recently published *IntCal20* and *Marine20* calibration curves[11,17]. For each dataset we used 50,000 MCMC iterations with 10,000 burn-in steps to infer the posterior dates of each layer of the sediments. The Poisson-Gamma model provides also an estimate of outlier probability for each radiocarbon date[13], and thus we ran the model iteratively excluding all dates with an outlier probability above 0.1 at each iteration. Table S2-S4 provides the final dates we used for each age depth model. We applied different Delta R corrections for marine dates depending on the location of the core. For the Pomaeho core we estimated a local Delta R of -213±52 using Reimar and Reimar[18] formula on two dates (KGM-OWd150387 and BETA-449188) recovered from the same sediment layer at 1,184 cm (see table S2). For the SSDP-102 sediment core we used a Delta R of -134±100 which is the the weighted mean between four published Delta R values from the southern coast (-94±22 and -71± 24[19], -296±35 and -253±45[20]) adjusted for the *Marine20* calibration curve.

LabCode	C14 Age	Depth (in cm)	Material	Included
KIA 19445	682±60	47	Bulk benthic foraminifera	Yes
KIA 19446	2090±25	305	Bulk benthic foraminifera	Yes
KIA 14913	1885±35	412	Bulk benthic foraminifera	Yes
KIA 19447	2630±20	509	Bulk benthic foraminifera	Yes

KIA 19448	4830±50	1050	Bulk benthic foraminifera	Yes
KIA 19449	4990±30	1125	Bulk benthic foraminifera	Yes
NZA10040	5715±81	1405	Lenticulina calcar (Linne)	Yes
NZA10041	7610±55	1623	Bulk benthic foraminifera	Yes
NZA10042	10858±80	2293	Bulk benthic foraminifera	Yes
NZA10757	11474±60	2310	Peaty Sediment	Yes

Table S2. Radiocarbon dates for the SSDP-102 sediments.

LabCode	C14 Age	Depth (in cm)	Material	Included
KGM-OCa150061	370±30	487	Shell (Potamocorbula sp.)	No
BETA-447816	1160±30	584	Shell (Potamocorbula sp.)	Yes
BETA-447818	1980±30	684	Bulk sediments	Yes
BETA-447819	2640±30	750	Bulk sediments	No
KGM-OCa150062	1990±30	831	Shell (Lacuna sp.)	Yes
BETA-447817	1910±30	871	Shell (Lacuna sp.)	No
BETA-457776	2750±30	891	Bulk sediments	Yes
BETA-457777	3700±30	931	Bulk sediments	Yes
BETA-447820	3600±30	970	Bulk sediments	Yes
BETA-457779	2760±30	1051	Bulk sediments	No
BETA-447821	6440±30	1105	Bulk sediments	No
BETA-457778	5040±30	1111	Bulk sediments	Yes
KGM-OWd150387	5880±40	1184	Wood fragments	Yes
BETA-449188	6230±30	1184	Bulk sediments	Yes

Table S3. Radiocarbon dates for the Pomaeho sediments.

LabCode	C14 Age	Depth (in cm)	Material	Included
KGM-IWd170355	1800±30	363	Plant fragments	Yes
KGM-IWd170356	2810±30	739	Plant fragments	Yes
KGM-IWd170357	4150±30	889	Plant fragments	Yes
KGM-IWd170358	6100±40	917	Plant fragments	Yes
KGM-IWd170359	7210±40	1416	Plant fragments	Yes
KGM-IWd170360	7900±40	1839	Plant fragments	Yes
KGM-IWd170361	7960±40	2060	Plant fragments	Yes

KGM-IWd170362	8030±40	2351	Plant fragments	Yes
KGM-IWd170363	8870±40	2892	Plant fragments	Yes1

Table S4. Radiocarbon dates for the GY-1 sediments.

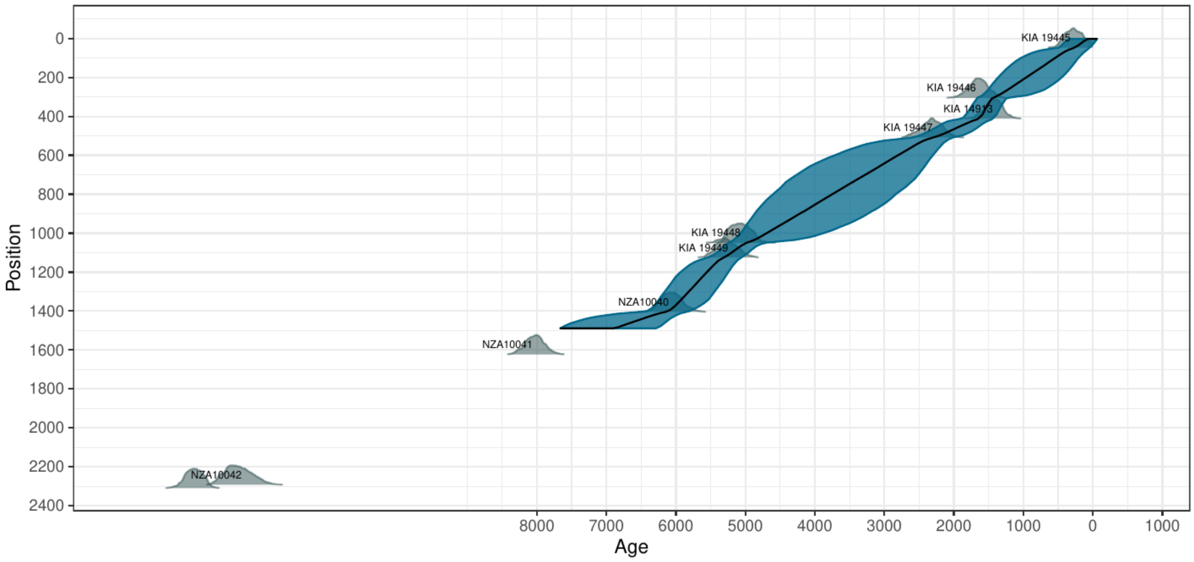

Figure S4. Age-depth model of the SSDP-102 sediment core.

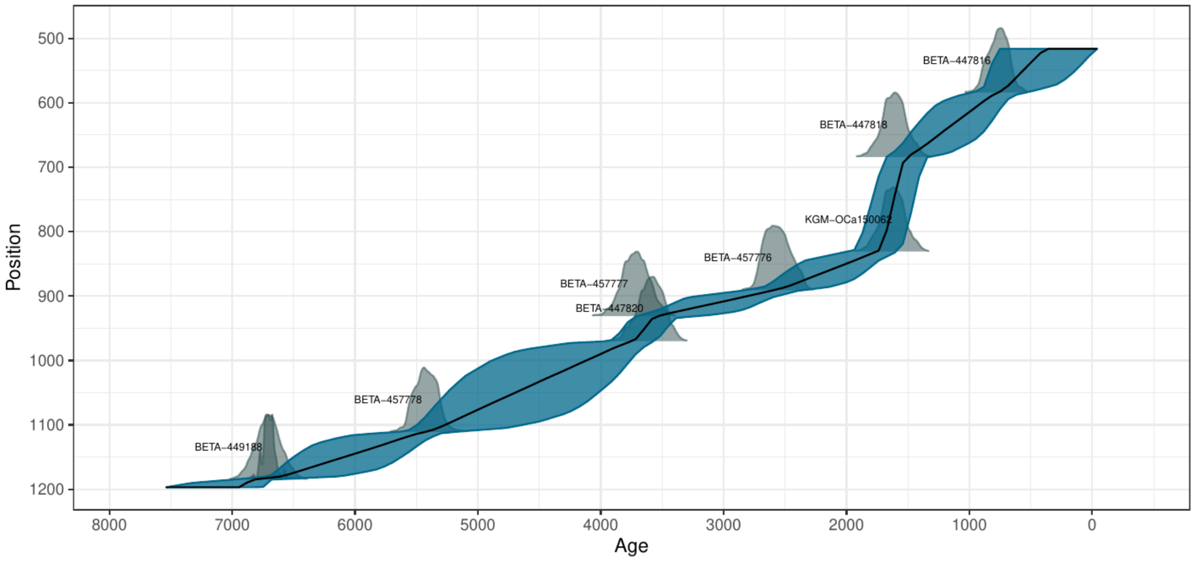

Figure S5. Age-depth model of the Pomacho site core.

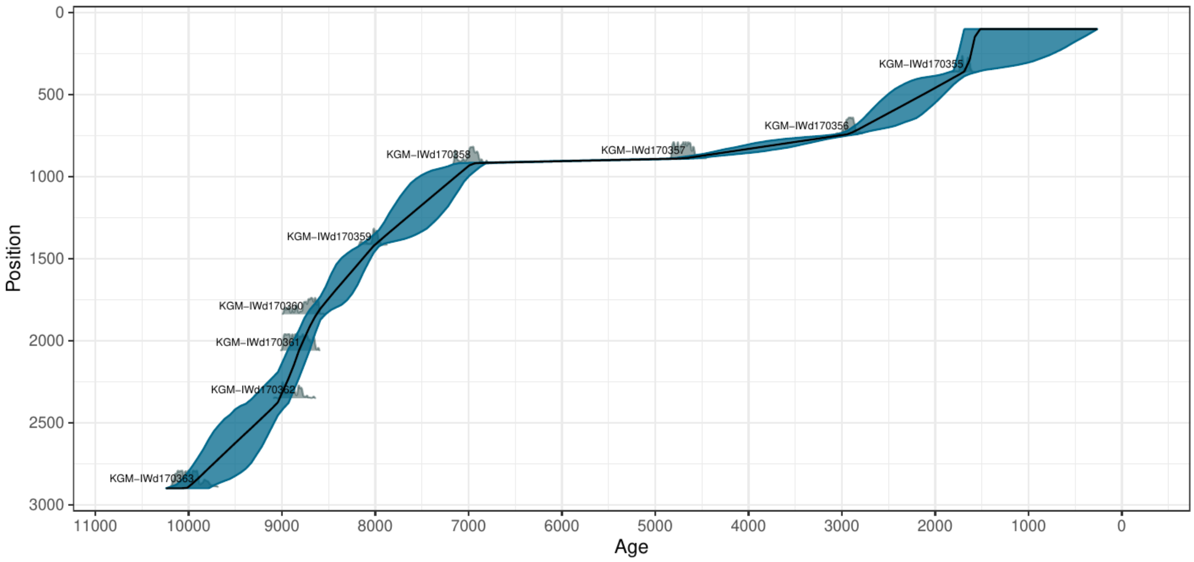

Figure S6. Age-depth model of the GY-1 sediment core.

For each dataset we have identified the start and end point of the largest cooling event within the temporal window of analysis (Fig. S7), namely between layer 1050cm and 1035cm in SSDP-102 (d_1 and d_2), between 1020 and 1017cm at Pomaeho (f_1 and f_2), and between 890 and 887 cm at GY-1 (g_1 and g_2), and extracted posterior samples (Fig. S8) to determine their chronology.

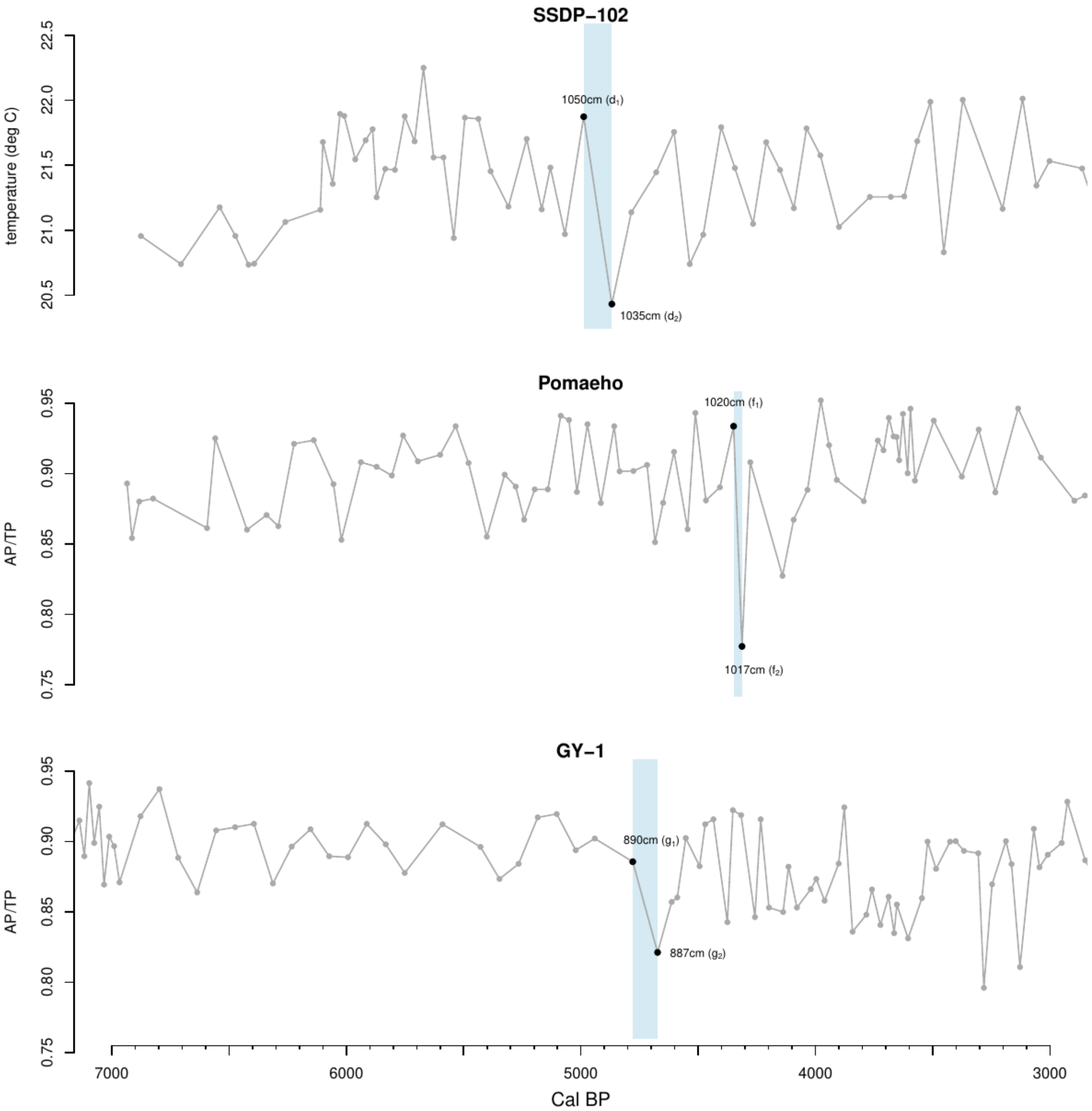

Figure S7. Climatic proxies and cooling events: alkenone-derived sea surface temperature reconstruction from the SSDP-102 sediment core (top)[14], ratio of arboreal pollen to total pollen from Pomaeho site (middle)[16], ratio of arboreal pollen to total pollen from the GY-1 sediment core (bottom)[15]

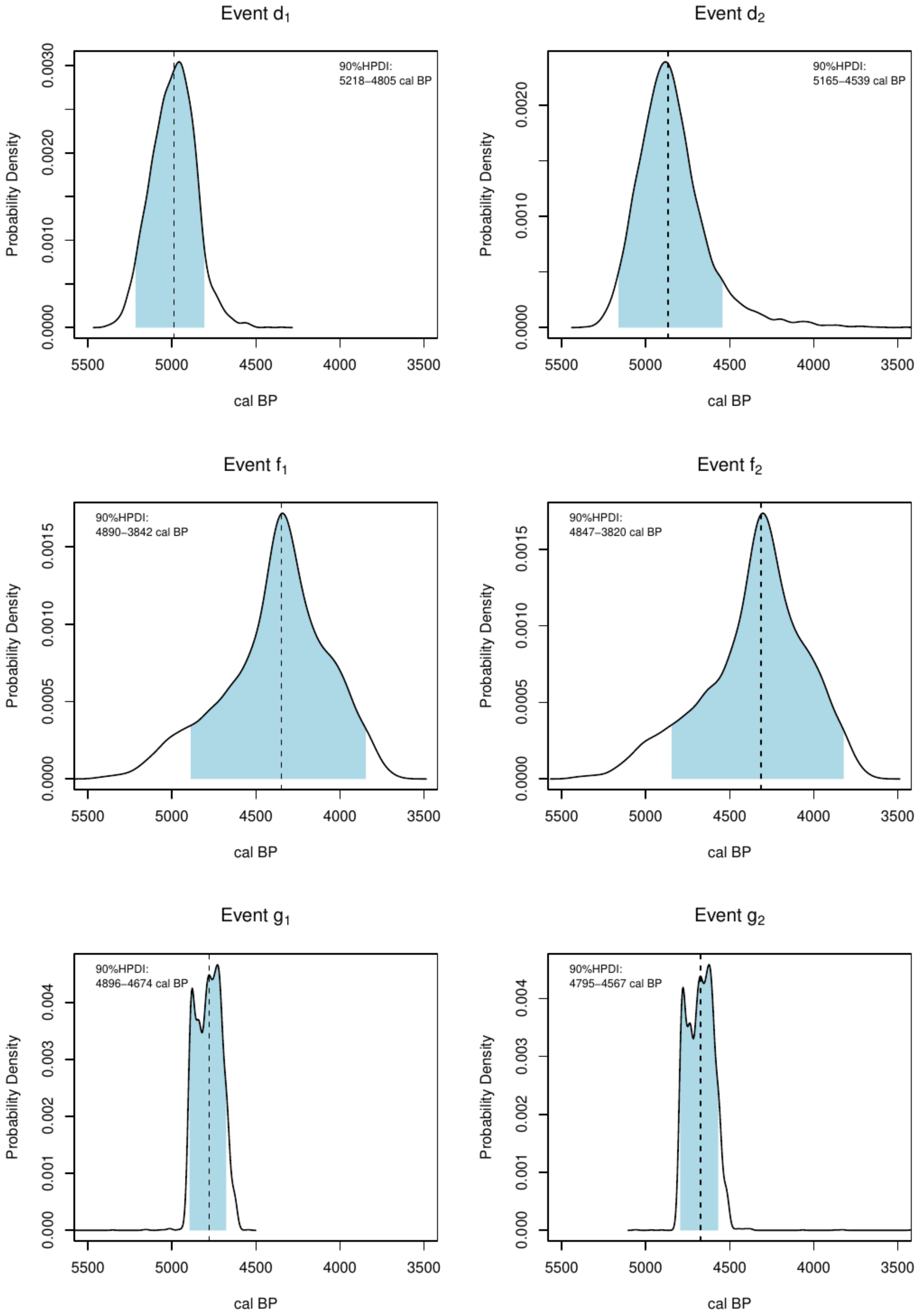

Figure S8. Posterior distribution of the start and end point of abrupt cooling events between 7,000 and 3,000 at SSDP-102 (d_1 and d_2), Pomaeho (f_1 and f_2), and GY-1 (g_1 and g_2).

5. Bayesian Modelling of Growth Rates and Change-points

We used the nimble[21,22] and nimbleCarbon[23] R packages to fit the following Bayesian model:

$$\theta_j \sim BoundedDoubleExponential(a, b, r_1, r_2, c)$$
$$x_j \sim Normal(\mu(\theta_j), \sigma_j)$$

where θ_j is the calendar date of the sample j, $\mu(\theta_j)$ is the corresponding ^{14}C age based on the IntCal20 calibration curve, σ_j is the root of the sum of the squares of the j's ^{14}C age error and the corresponding error in the calibration curve, and x_j is the observed ^{14}C age of j. The *BoundedDoubleExponential* model is generalised bernoulli distribution where the probability of observing a sample from the calendar year (in BP) t is given by the probability mass function:

$$p_{t=a-i} = \frac{(1+r)^i}{\sum_{i=0}^{a-b}(1+r)^i}$$

where

$$r = \begin{cases} r_1 \; if \;\; t > c \\ r_2 \; if \;\; t \leq c \end{cases}$$

and with $p_t = 0$ when $t > a$ and when $t < b$. Here we are interested in estimating from the *BoundedDoubleExponential* the growth rates r_1 and r_2, and their change point c. To achieve this we used the following constants and priors:

$$a \;=\; 7000$$
$$b \;=\; 3000$$
$$r_1 \sim Normal(\mu = 0, \sigma = 0.0004)$$
$$r_2 \sim Norma(\mu = 0, \sigma = 0.0004)$$
$$c \sim TruncatedNormal(a = 3000, b = 7000, \mu = 5000, \sigma = 1000)$$

which ensures a wide range of realistic patterns (see Fig. S9) with growth rates comparable to those observed from other SPDs and a weakly informative prior for c reducing the probability of the shift in growth rate at the extremes of the window of analysis.

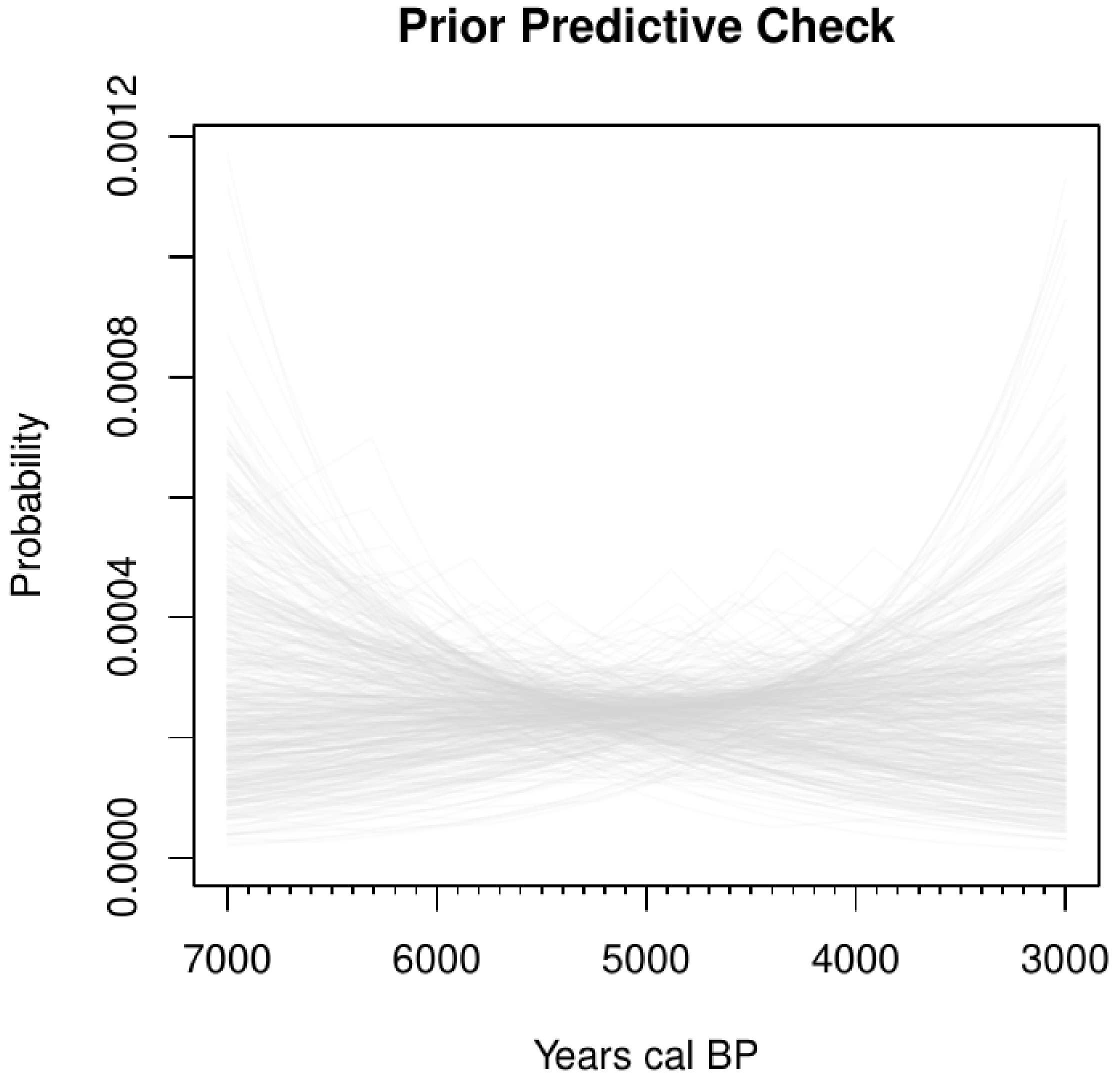

Figure S9. Prior predictive check of the bounded double exponential growth model.

Posterior samples for the coastal, inland, and combined datasets were obtained using *nimble*'s Metropolis-Hastings adaptive random-walk sampler, with three chains of 100,000 iterations, a burn-in of 10,000 steps, and a thinning interval of 6 iterates (see Fig. S10 for trace plots). The 90% highest posterior density interval, Gelman-Rubin convergence diagnostic ($\hat{R}$), and the effective sample size of the three parameters for the three models are shown on table S5, while the fitted model and marginal posterior distributions are shown on Fig. S11 and S12.

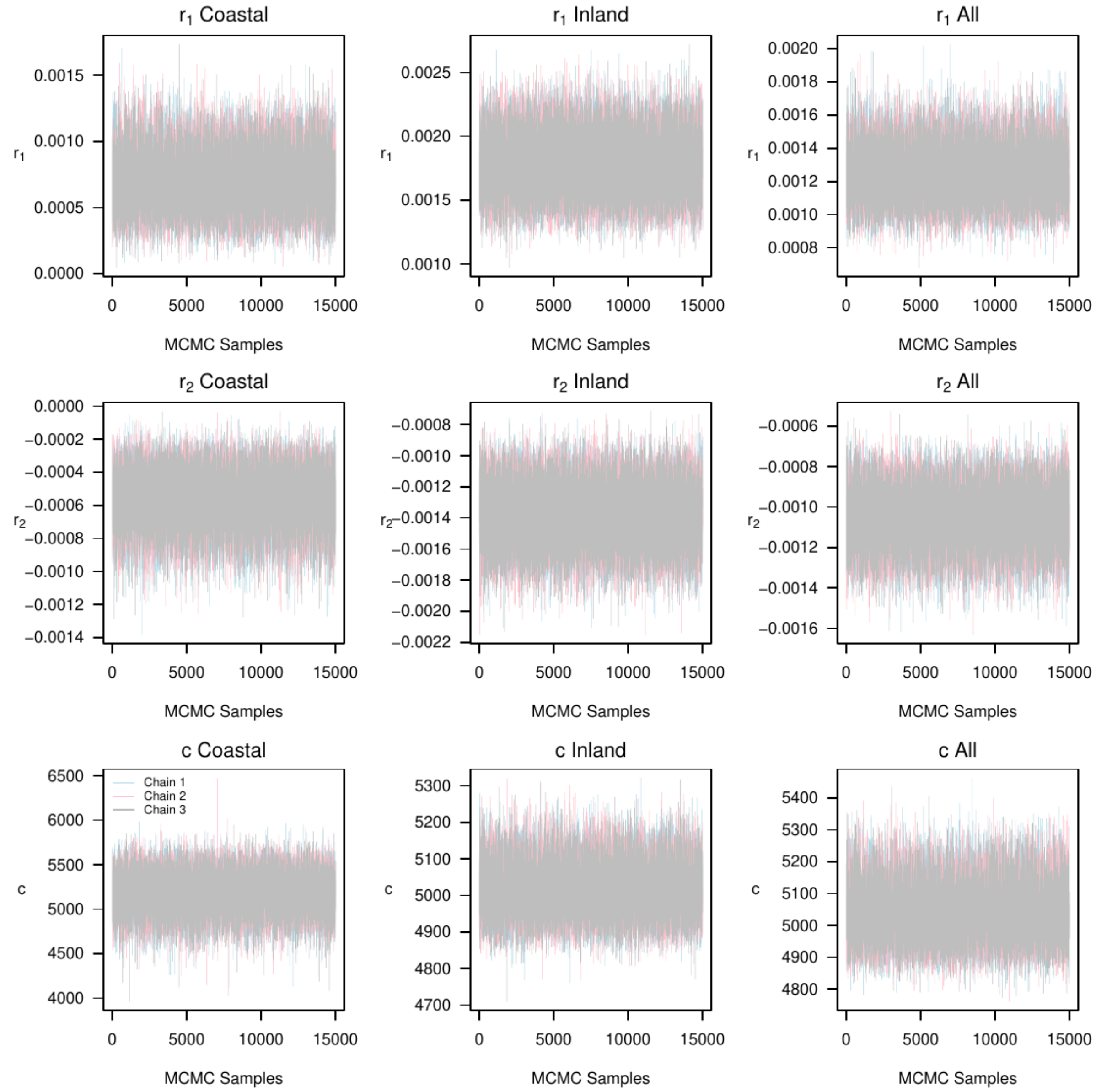

Figure S10. MCMC Trace plots for the coastal, inland, and combined datasets.

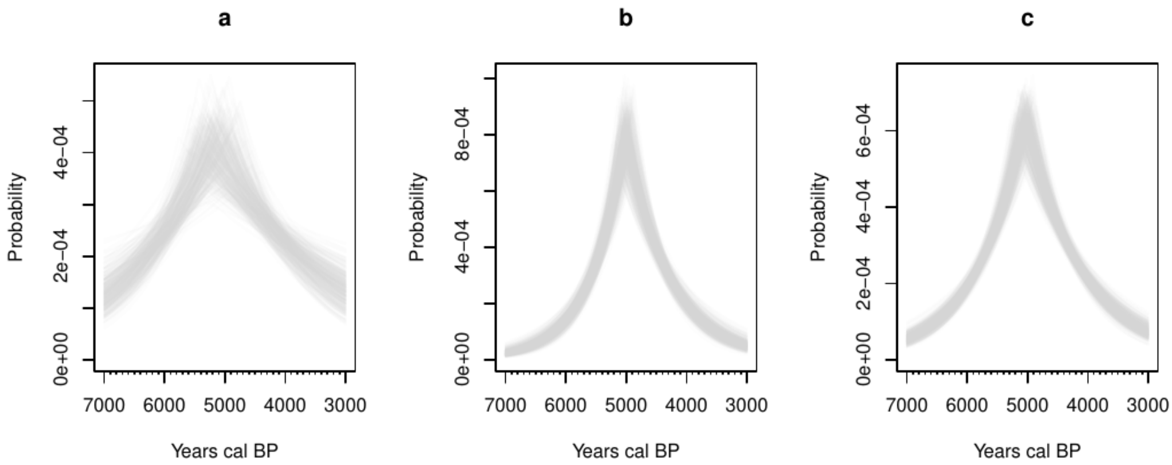

Figure S11. Fitted bounded double exponential growth model for the coastal, inland, and combined datasets.

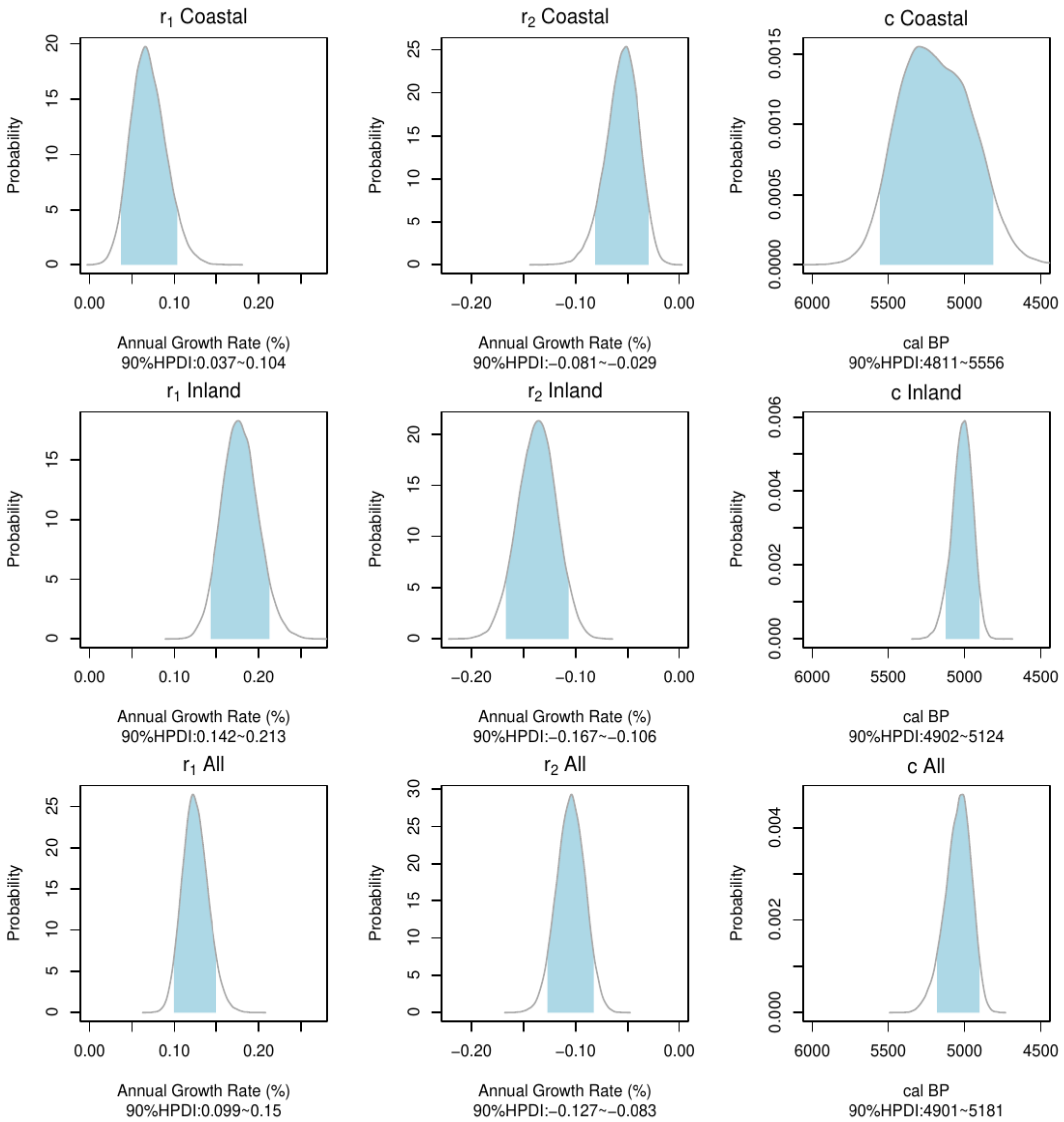

Figure S12. Marginal posterior distribution of the growth rates (r_1 and r_2) and change points (c) for the coastal, inland, and combined datasets.

Parameter	Data	90% HPD	ESS	$\hat{R}$
r_1	Inland	0.00136 ~ 0.00221	25047.20	1.000
	Coastal	0.00031 ~ 0.00111	15594.01	1.001
	Combined	0.00095 ~ 0.00156	16786.76	1.001
r_2	Inland	-0.00174 ~ -0.00102	23394.61	1.000
	Coastal	-0.00087 ~ -0.00025	17369.63	1.000
	Combined	-0.00132 ~ -0.00079	16393.88	1.000
c	Inland	5151 ~ 4886 cal BP	17410.83	1.001
	Coastal	5614 ~ 4743 cal BP	11101.37	1.001
	Combined	5215 ~ 4879 cal BP	11016.00	1.000

Table S5. MCMC summary statistics.

To assess the goodness-of-fit of the growth model we carried out a posterior predictive check using the *postPredSPD*() function in the *nimbleCarbon* R package. The function generates an envelope of SPDs according to the fitted model and visualises time-intervals where the observed SPD showed a higher or lower density of radiocarbon dates. We generated the fitted model envelope of the three datasets using a 95% percentile interval computed from 500 simulated SPDs using randomly drawn parameter combinations from the joint posterior distribution (Fig. S13).

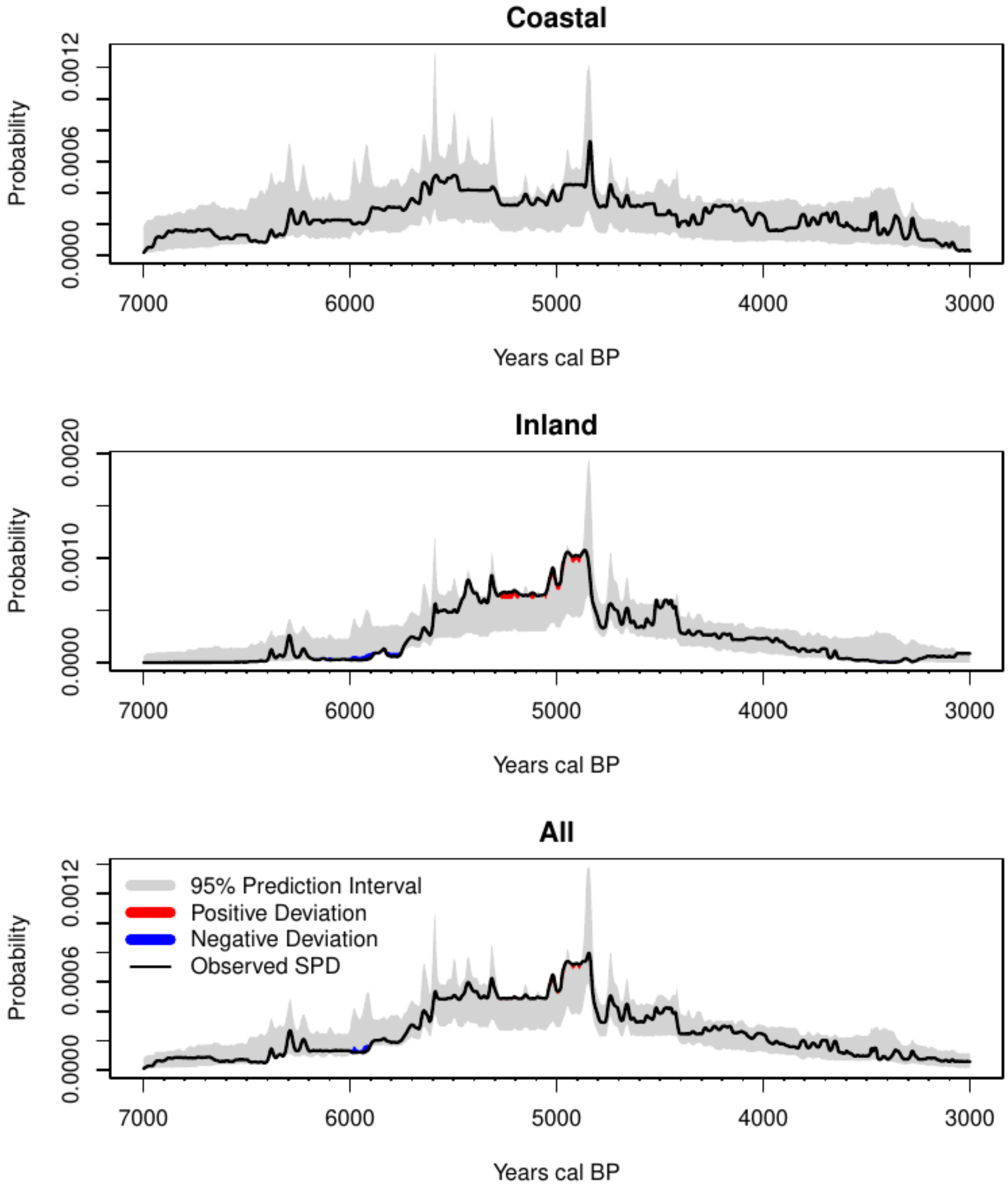

Figure S13. Posterior predictive checks of the fitted models

References

1. Bae, K., Bae, C. J. & Kim, J. C. Reconstructing Human Subsistence Strategies During the Korean Neolithic: Contributions from Zooarchaeology, Geosciences, and Radiocarbon Dating. *Radiocarbon* **55**, 1350–1357 (2013).
2. So, S. Y. KOR Neolithic Radiocarbondate (INTCAL13 calibrated,Mar2016). *Korean Neolithic Research Society* (2016).
3. Gimhae National Museum. *Bibong-ri Site*. (Gimhae National Museum, 2008).
4. Pusan National University Museum. *Gimhae Sugari Paechong I*. (Pusan National University Museum, 1981).
5. The Ulsan Institute of Cultural Properties. *Pusan Gadong Paechong*. (The Ulsan Institute of Cultural Properties, 2014).
6. Center of History & Culture, Gyeongnam Development Institute. *The Excavation Report of Jungnim-dong Site, Busan*. (Center of History & Culture, Gyeongnam Development Institute, 2009).
7. Crema, E. R. & Bevan, A. INFERENCE FROM LARGE SETS OF RADIOCARBON DATES: SOFTWARE AND METHODS. *Radiocarbon* **63**, 23–39 (2021).
8. R Core Team. *R: A language and environment for statistical computing*. (R Foundation for Statistical Computing, Vienna, Austria, 2021).
9. Ester, M., Kriegel, H. P., Sander, J. & Xu, X. A Density-Based Algorithm for Discovering Clusters in Large Spatial Databases with Noise. in *Proceedings of 2nd International Conference on Knowledge Discovery and Data Mining (KDD-96)* (eds. Simoudis, E., Han, J. & Fayyad, U.) 226–231 (Portland: AAAI Press, 1996).
10. Timpson, A. *et al.* Reconstructing regional population fluctuations in the European Neolithic using radiocarbon dates: a new case-study using an improved method. *Journal of Archaeological Science* **52**, 549–557 (2014).
11. Reimer, P. J. *et al.* The IntCal20 Northern Hemisphere Radiocarbon Age Calibration Curve (0–55 cal kBP). *Radiocarbon* **62**, 725–757 (2020).

12. Crema, E. R., Habu, J., Kobayashi, K. & Madella, M. Summed Probability Distribution of 14C Dates Suggests Regional Divergences in the Population Dynamics of the Jomon Period in Eastern Japan. *PLoS ONE* **11**, e0154809 (2016).

13. Haslett, J. & Parnell, A. A simple monotone process with application to radiocarbon-dated depth chronologies. *Journal of the Royal Statistical Society: Series C (Applied Statistics)* **57**, 399–418 (2008).

14. Kim, J. H. *et al.* North Pacific and North Atlantic sea-surface temperature variability during the Holocene. *Quaternary Science Reviews* **23**, 2141–2154 (2004).

15. Park, J. J. *et al.* Abrupt Holocene climate shifts in coastal East Asia, including the 8.2 ka, 4.2 ka, and 2.8 ka BP events, and societal responses on the Korean peninsula. *Scientific Reports* **9**, 10806 (2019).

16. Constantine, M., Kim, M. K. & Park, J. J. Mid- to late Holocene cooling events in the Korean Peninsula and their possible impact on ancient societies. *Quaternary Research* **92**, 98–108 (2019).

17. Heaton, T. J. *et al.* Marine20—The Marine Radiocarbon Age Calibration Curve (0–55,000 cal BP). *Radiocarbon* **62**, 779–820 (2020).

18. Reimer, R. & Reimer, P. An Online Application for ΔR Calculation. *Radiocarbon* **59**, 1623–1627 (2016).

19. Kim, H., Lee, H. & Lee, G. NEW MARINE RESERVOIR CORRECTION VALUES (ΔR) APPLICABLE TO DATES ON NEOLITHIC SHELLS FROM THE SOUTH COAST OF KOREA. *Radiocarbon* **63**, 1287–1302 (2021).

20. Kong, G. & Lee, C. Marine reservoir corrections (ΔR) for southern coastal waters of Korea. *The Sea: Journal of the Korean Society of Oceanography* **10**, 124–128 (2005).

21. de Valpine, P. *et al.* Programming with models: writing statistical algorithms for general model structures with NIMBLE. *Journal of Computational and Graphical Statistics* **26**, 403–413 (2017).

22. de Valpine, P. *et al. NIMBLE: MCMC, Particle Filtering, and Programmable Hierarchical Modeling*. (R package version 0.11.1, 2021).

23. Crema, E. R. *nimbleCarbon: Models and Utility Functions for Bayesian Analysis of Radiocarbon Dates with NIMBLE (v.0.1.0)*. (2021).
